# Sex differences in the association of cardiometabolic risk scores and blood pressure measurements with white matter hyperintensities in diverse older adults—HABS-HD

**DOI:** 10.3389/fnagi.2025.1607646

**Published:** 2025-08-04

**Authors:** Cellas A. Hayes, Raul Vintimilla, Soumilee Chaudhuri, Michelle C. Odden

**Affiliations:** ^1^Department of Epidemiology and Population Health, Stanford University School of Medicine, Palo Alto, CA, United States; ^2^Institute for Translational Research, University of North Texas Health Science Center, Fort Worth, TX, United States; ^3^Department of Radiology and Imaging Sciences, Center for Neuroimaging, Indiana University School of Medicine, Indianapolis, IN, United States; ^4^Indiana Alzheimer’s Disease Research Center, Indiana University School of Medicine, Indianapolis, IN, United States

**Keywords:** aging, cardiometabolic risk factors, sex differences, vascular brain injury, white matter hyperintensities, blood pressure, diastolic blood pressure

## Abstract

**Introduction:**

We aimed to determine whether cardiometabolic risk factors and blood-pressure (BP) metrics were differentially associated with white matter hyperintensities volume (WMHV) in males versus females in the Health and Aging Brain Study–Health Disparities.

**Methods:**

We analyzed 3,585 community-dwelling adults (2,207 females) from non-Hispanic White, non-Hispanic Black, and Hispanic groups who underwent BP measurement and WMHV quantification. Linear regression models assessed (i) individual risk factors (diabetes, hypertension, dyslipidemia, obesity, tobacco dependence), (ii) a composite risk score, and (iii) four BP metrics (systolic, diastolic, pulse pressure, mean arterial pressure), each including a sex-interaction term and adjusting for age, education, race/ethnicity, and scanner. A second BP model also controlled for all five risk factors.

**Results:**

Diabetes (β = 0.46, 95% CI 0.28–0.64), hypertension (β = 0.47, 0.30–0.64), and higher composite risk (β = 0.19, 0.12–0.26) were associated with greater WMHV. Diastolic BP (β = 0.18, 0.11–0.26) and mean arterial pressure (β = 0.14, 0.07–0.21) related to larger WMHV, with diastolic BP remaining significant after full adjustment (β = 0.14, 0.07–0.22). No sex interactions survived correction.

**Discussion:**

These findings underscore the importance of aggressive cardiometabolic and BP control, particularly diastolic BP, to mitigate WMHV in both sexes.

## Introduction

Cardiometabolic risk factors such as diabetes, hypertension, dyslipidemia, obesity, and tobacco use increase the likelihood of cardiovascular disease, cerebrovascular disease, and Alzheimer’s disease (Santos et al., 2017). Previous research indicates that males exhibit a higher prevalence of these risk factors in mid-life, whereas females are more affected in late life (Appelman et al., 2015; Samad et al., 2008). While the interaction between cardiometabolic risk factors and sex on neurodegenerative outcomes like cognition has been studied extensively, fewer investigations have explored their interactive effects on white matter hyperintensities volume (WMHV), a key marker of cerebrovascular and small vessel disease (Patel and Edison, 2024).

WMHV, detected through magnetic resonance imaging (MRI), represent damage to small blood vessels in the brain and are strongly associated with an increased risk of stroke, dementia, and other neurological disorders (Prins and Scheltens, 2015). Hypertension, diabetes, and obesity are well-documented contributors to WMHV development, but their effects may differ by biological sex (henceforth sex) due to differences in vascular physiology and hormonal influences. Hypertension is arguable the strongest risk factor for WMHV independent of age (Jeerakathil et al., 2004; Scharf et al., 2019). A previous limitation has been the lack of differentiating the roles of blood pressure hemodynamics [systolic blood pressure (SBP), diastolic blood pressure (DBP), and derived measures such as pulse pressure (PP) and mean arterial pressure (MAP)] on WMHV (de Leeuw et al., 2002; Hayes et al., 2025). This gap underscores the need for a nuanced understanding of how specific blood pressure components contribute to WMHV and whether these relationships vary between males and females.

Previously, SBP and a history of hypertension were associated with total WMHV in males, but not females, whereas a vascular composite was more strongly associated with total WMHV in females than males (Morrison et al., 2023). In a study from Germany, hypertension and current smoking showed stronger associations with more extensive WMHV in females compared to males (Bonberg et al., 2022). Recent findings from the Framingham Heart Study show evidence that higher Framingham stroke risk scores and SBP are associated with increased WMHV in females, and these findings were significant when controlling for different menopause factors (Yau et al., 2024). However, it is unclear whether these findings from Yau and colleagues in the Framingham Heart Study apply to racially and ethnically diverse older adults. In our recent findings, we showcase that hypertension is a risk factor but not diabetes, dyslipidemia, obesity, or tobacco dependence in diverse older adult females (Hayes et al., 2025). Moreover, both hypertension and diabetes had interactions with Hispanic racial ethnicity for less and more WMHV, respectively (Hayes et al., 2025). Although our study found main effects of all blood pressure hemodynamics and no interactions with racial ethnicity, this study was only limited to a subsample of female participants.

The primary objective of this study was to identify whether cardiometabolic conditions and blood-pressure measurements explain WMHV differently in males versus females in a multi-ethnic cohort, the Health and Aging Brain Study - Health Disparities (HABS-HD). By focusing on sex-specific interactions and differences, this research aims to inform the development of tailored prevention strategies to promote brain health and reduce disparities in vascular-related neurological outcomes.

## Materials and methods

### Study design

The Health and Aging Brain Study—Health Disparities (HABS-HD) (established in 2017) was formerly known as The Health and Aging Brain among Latino Elders (HABLE) (conducted between 2012 and 2017) (O’Bryant et al., 2021). HABS-HD is a longitudinal research study at the Institute for Translational Research in the University of North Texas Health Science Center (O’Bryant et al., 2021). To date, HABS-HD represents the largest comprehensive study of Alzheimer’s disease that includes diverse participants including non-Hispanic White (NHW), non-Hispanic Black (NHB), and Hispanic older adults in the United States. The focus of the HABS-HD study is to understand and eliminate health disparities among underrepresented populations which were previously not a major priority in Alzheimer’s disease longitudinal research cohorts.

The HABS-HD study inclusion criteria has been extensively described elsewhere (O’Bryant et al., 2021). Briefly, all HABS-HD participants undergo background/demographic questionnaires, comprehensive neuropsychological testing, clinical fluid laboratory assessments including blood draws, brain magnetic resonance imaging (MRI) scans, positron emission tomography (PET) scans (amyloid and tau), and functional exams (O’Bryant et al., 2021). Participants qualify for HABS-HD if they (1) self-identify as NHW, African American, or Hispanic; (2) are willing to provide a blood sample; (3) are 30 years of age or older (previously 50); (4) agree to undergo MRI and PET imaging; (5) are fluent in English or Spanish; and (6) can provide reliable information.

HABS-HD exclusion criteria is as follows: (1) a diagnosis of type 1 diabetes; (2) an uncontrolled inflammatory disorder at the time of screening; (3) a current or recent (within 12 months) malignancy other than non-melanoma skin cancer or non-metastatic prostate cancer; (4) a severe psychiatric illness likely to compromise cognitive performance (depression and anxiety are not exclusionary); (5) a traumatic brain injury with loss of consciousness in the past 12 months; (6) current alcohol or substance abuse; (7) a serious medical condition that can impair cognition, such as end-stage renal disease or epilepsy; (8) chemotherapy or radiotherapy in the preceding 12 months; or (9) any medical device or condition that precludes safe MRI or PET imaging.

The de-identified HABS-HD participant data are available upon request for research purposes (see “Data availability” section). All protocols and participants written informed consent was obtained and approved by the North Texas Regional Institutional Review Board. The HABS-HD study follows the Declaration of Helsinki ethical guidelines.

### Demographic questionnaire

HABS-HD participants self-reported racial ethnic backgrounds as NHW, NHB, and Hispanic. The demographics questionnaire also collected the age of each participant at the visit and the number of years of education that they had acquired. The HABS-HD questionnaire asks each participant, “What is your gender?” with two optional responses, male or female. Therefore, the true question reflects gender with a binary outcome. In this study, we refer to gender as sex given that the question only had two options available and not responding was prohibited. In addition, several other HABS-HD studies report sex with male/female instead of gender (Hayes et al., 2025; King et al., 2022; Vintimilla et al., 2021). Aligning with previous studies, we refer to the binary self-reported gender questionnaire response as sex throughout (Hayes et al., 2025; King et al., 2022; Vintimilla et al., 2021).

### Cardiometabolic risk factors

Consensus meetings and review were used to determine the diagnoses of hypertension, diabetes, dyslipidemia, and tobacco dependence. Hypertension was defined by a self-reported medical diagnosis, use of antihypertensive medications, and/or an average of two blood pressure readings ≥ 140/90 mmHg. Diabetes was identified through a self-reported medical diagnosis, current use of insulin or oral hypoglycemic medications, and/or an HbA1c level ≥ 6.5%. Dyslipidemia was characterized by a self-reported diagnosis of elevated cholesterol or triglycerides, use of lipid-lowering medications, total cholesterol levels ≥ 200 mg/dL, and/or triglycerides ≥ 150 mg/dL. We defined obesity through body mass index (30.0 or higher). Clinical consensus also determined whether participants were tobacco dependent using psychological information (i.e., participant self-report; individuals who reported current smoking during interviews were classified as tobacco dependent). The variables from the HABS-HD data request that were used is as follows: CDX_Diabetes, CDX_Hypertension, CDX_Dyslipidemia, CDX_Tobacco, and OM_BMI.

### Blood pressure measurements

Resting SBP and DBP were measured using the traditional approach using a sphygmomanometer twice and the average was used for analyses in this study. PP was determined by the difference between SBP and DBP calculated as PP = SBP −DBP. PP reflects the force exerted by the heart with each contraction and is often used to assess arterial stiffness and vascular health. MAP was calculated using the formula: MAP = (1/3) × (DBP × 2 + SBP) (Yau et al., 2024). MAP provides a weighted average of blood pressure over the course of a heartbeat, offering a critical indicator of tissue perfusion and overall cardiovascular function. Systolic, diastolic, pulse pressure, and mean arterial pressure values were scaled by dividing by 10 to aid interpretability of model coefficients, representing per 10 mmHg increments. Physiologically implausible blood pressure values (i.e., < 0) were treated as missing prior to analysis.

### Neuroimaging MRI

The neuroimaging methodology has been described in the original HABLE study, and the protocols were adopted in 2017 at the start of HABS-HD (O’Bryant et al., 2021). MRI data were collected for all participants using a standardized protocol on a 3T scanner (Siemens Healthineers AG, Erlangen, Germany). Two scanners were used: (1) Skyra and (2) Vida. The imaging protocol included T1-weighted magnetization-prepared rapid acquisition gradient echo (MPRAGE) sequences with a repetition time of 2,300 ms, echo time of 2.93 ms, field of view of 270 mm, a matrix of 256, slice thickness of 1.2 mm, and voxel dimensions of 1.1 mm × 1.1 mm × 1.2 mm. Additionally, T2-weighted fluid-attenuated inversion recovery (FLAIR) sequences were acquired with a repetition time of 4,800 ms, echo time of 441 ms, inversion time of 1,650 ms, field of view of 256 mm, a matrix of 256, slice thickness of 1.2 mm, and voxel dimensions of 1.0 mm × 1.0 mm × 1.2 mm. All images were obtained in the sagittal plane.

### WMHV

WMHV was measured from the MPRAGE and FLAIR images using the Statistical Parametric Mapping Lesion Segmentation Toolbox Lesion Growth Algorithm^1^ (Schmidt et al., 2012). The raw WMHV were strongly right-skewed with occasional zero values. We first stabilized the WMHV distribution by adding a tiny constant (1 × 10^–6^) and taking the natural logarithm. We then adjusted these log-transformed volumes for intracranial volume (ICV)—measured via high-resolution T1 segmentation—using a linear regression model, consistent with prior HABS-HD and neuroimaging protocols (Hayes et al., 2025; King et al., 2022; O’Brien et al., 2011). Model fit was assessed with a residuals-versus-fitted plot (Supplementary Figure 1A). Applying a 1.5 × IQR rule to the residuals, we flagged and removed 248 outlying participants (≈6% of the sample). Finally, we refit the log-WMH∼ICV model on the cleaned dataset, inspected its residuals (Supplementary Figure 1B), and saved the resulting residuals for all downstream analyses. The resulting extracted residuals represent WMHV independent of ICV and served as the primary volumetric outcome throughout this study. The final sample size post-excluding WMHV outliers was 3,585. The model diagnostics indicated satisfactory homoscedasticity and no influential points by plotting the fitted versus the residuals. In this study, WMHV is represented by an ICV-adjusted, log-scaled WMHV residuals free of extreme outliers.

### Statistical analyses

#### Sample characteristics comparisons

Descriptive analyses were conducted to compare demographic, cardiometabolic, and cognitive characteristics between males and females. Continuous variables were summarized using means and standard deviations (SD), while categorical variables were presented as raw counts and percentages. Independent two-sample *t*-tests were used for continuous variables, and chi-square tests of independence were conducted to evaluate differences in proportions between males and females.

#### Cardiometabolic risk score

To calculate the cardiometabolic disease risk score, principal component analysis (PCA) was utilized to create a composite variable summarizing the 5 key risk factors. The harmonized risk score was derived from the categorical clinical consensus derived cardiovascular risk factors: diabetes, hypertension, dyslipidemia, and tobacco dependence along with obesity defined by BMI. Age, sex, and race were not included in the risk score because we controlled for them in the statistical models; therefore, we did not want to over adjust the findings related to the risk score.

Cardiometabolic burden score was a continuous score derived from PCA for mixed data. Five binary indicators—obesity (body-mass-index ≥ 30 kg/m^2^), diabetes, hypertension, dyslipidemia, and tobacco dependence. Each variable included in the model had two levels. We used the PCAmixdata package (v 3.1) in R 4.3.1. The five binary risk factors (obesity, diabetes, hypertension, dyslipidemia, and tobacco dependence) were moderately correlated (average φ = 0.48; max = 0.68). The PCA model produced a first component (PC1) with an eigen-value of 1.40 that explained 28.1% of total inertia, whereas the other components explained less variance (PC2 = 20.5%, PC3 = 19.6%, PC4 = 16.5%, PC5 = 15.3%; see scree plot in Supplementary Figure 2). Diagnostic tests confirmed factorability (Bartlett’s χ^2^_10_ = 436.3, *p* < 0.001) and acceptable sampling adequacy for binary data (overall KMO = 0.57; item-level KMOs = 0.42–0.60).

The scores derived for PC1 were extracted and assigned as the cardiometabolic risk score for each participant. To facilitate interpretability, PC1 scores were inverted so that higher values corresponded to greater cardiometabolic risk. The risk score distribution was approximately symmetric (median = −0.13, interquartile range = 1.74, range = −2.18 to 2.11), supporting its use as a continuous exposure in subsequent regression models. To generate the score, we followed methodology previously used for the Cardiovascular Harmonization Workflow and the Alzheimer’s Disease Sequencing Project Phenotype Harmonization Consortium (ADSP-PHC) (Hohman, 2023; Lee et al., 2023).

#### Multivariable linear regression

WMHV was analyzed as the natural logarithm of volumetric WMHV residuals after adjustment for ICV (see Imaging methods). Multivariable linear-regression models were fitted to quantify associations between cardiometabolic risk factors and WMHV, with an interaction term with sex.

For each risk factor (diabetes, hypertension, dyslipidemia, obesity, and tobacco dependence), we estimated the main effect and the sex × risk-factor interaction term while adjusting for age (years), education (years), self-identified racial ethnicity (NHW, NHB, and Hispanic), and MRI scanner. Second, the continuous cardiometabolic risk score derived from PCA (higher values indicating greater risk) was evaluated in an otherwise identical model that also included the sex × risk-score interaction. Third, four blood-pressure (BP) metrics [systolic BP, diastolic BP, pulse pressure (PP), and mean arterial pressure (MAP)] were analyzed. Each BP variable was divided by 10 so that regression coefficients represent the change in WMHV per 10-mmHg increments. Two BP model specifications were fitted: a base model controlling for the covariates listed above and an extended model that additionally adjusted for the five individual cardiometabolic risk factors to assess the independence of BP associations.

Because twelve hypotheses for the individual risk factors and risk score (six main effects and six interactions) and eight *a priori* hypotheses were tested for the BP metrics (four main effects and four sex interactions), two family-wise Bonferroni corrections were applied (α/12 = 0.004 for risk-factor models; α/8 = 0.006 for BP models). Nominal two-sided *p*-values are reported alongside Bonferroni-adjusted *p*-values; results surpassing the corrected threshold remain highlighted.

Regression coefficients (β) with 95% confidence intervals (CI) are reported throughout, where positive β values indicate greater WMHV associations. All regression models were restricted to participants with complete data on the respective outcome, exposure, and covariates. No missing data imputation techniques were applied. The fitted versus residuals of all models are shown in Supplementary Figure 3. Variance inflation factors (VIFs) were calculated using the car:vif() function for all models to formally assess multicollinearity (Supplementary Table 1). Adjusted VIFs *GVIF*∧[1/(2*Df)] were reported to account for categorical variables and interaction terms. In the individual risk-factor models (including sex interactions), adjusted VIFs ranged from 1.01 to 2.21. For the blood pressure models (SBP, DBP, PP, MAP), adjusted VIFs for covariates and blood pressure terms ranged from 1.06 to 1.79. Interaction terms between sex and blood pressure variables showed moderately elevated VIFs (range: 4.01 to 8.53). In the fully adjusted blood pressure models, which included five cardiovascular risk factors, VIFs for main effects remained low (range: 1.02 to 1.83), while interaction terms showed higher VIFs (range: 7.56 to 8.54), largely due to inherent collinearity between continuous BP terms and sex interactions. However, all VIFs remained below the conservative threshold of 10, indicating no problematic multicollinearity. Therefore, no variables were removed, and PCA was not applied. The full R scripts are provided in the Supplementary materials.

All analyses were conducted in R version 4.3.1.

## Results

Table 1 summarizes the demographic, cardiometabolic, BP, and WMHV characteristics of the 3,585 participants stratified by sex. On average, males were slightly older than females (66.1 ± 8.5 vs. 65.0 ± 8.7 years, *p* < 0.001) and reported more years of education (13.8 ± 4.3 vs. 13.2 ± 4.2 years, *p* < 0.001). The distribution of racial ethnicity differed between sexes (*p* < 0.001): Hispanic individuals comprised 39.6% of females but 32.3% of males, whereas NHW participants were more common among males (40.5%) than females (32.0%). With respect to cardiometabolic risk factors, hypertension was more prevalent in males than females (70.9% vs. 63.2%, *p* < 0.001), whereas obesity was slightly more common among females (49.4% vs. 44.6%, *p* = 0.005). Tobacco dependence was more than twice as frequent in males (11.2%) as in females (5.3%, *p* < 0.001). The composite cardiometabolic risk score was marginally higher in males (0.05 ± 1.2) than in females (−0.01 ± 1.2, *p* = 0.04). A history of clinically diagnosed cardiovascular disease was also more common in males (9.7%). All four blood-pressure metrics were significantly higher in males.

**TABLE 1:** Descriptive characteristics of the demographics, cardiometabolic risk factors, blood pressure measurements, and white matter hyperintensities of the overall sample stratified by sex.

Variable	Male (*N* = 1,378)	Female (*N* = 2,207)	*p*-value	Overall (*N* = 3,585)
Age (years) mean (SD)	66.1 (8.5)	65.0 (8.7)	< 0.001	65.4 (8.6)
Education (years) mean (SD)	13.8 (4.3)	13.2 (4.2)	< 0.001	13.4 (4.2)
Racial ethnicity *n* (%)			< 0.001	
Non-Hispanic White	558 (40.5)	706 (32.0)		1,264 (35.3)
Non-Hispanic Black	375 (27.2)	628 (28.5)		1,003 (28.0)
Hispanic	445 (32.3)	873 (39.6)		1,318 (36.8)
Diabetes *n* (%)	368 (26.7)	529 (24.0)	0.07	897 (25.0)
Hypertension *n* (%)	977 (70.9)	1,395 (63.2)	< 0.001	2,372 (66.2)
Missing *n* (%)	0 (0)	1 (0.0)		1 (0.0)
Dyslipidemia *n* (%)	956 (69.4)	1,510 (68.4)	0.57	2,466 (68.8)
Obesity *n* (%)	615 (44.6)	1,090 (49.4)	0.005	1,705 (47.6)
Missing *n* (%)	6 (0.4)	14 (0.6)		20 (0.6)
Tobacco dependence *n* (%)	155 (11.2)	118 (5.3)	< 0.001	273 (7.6)
Cardiometabolic risk score mean (SD)	0.05 (1.2)	−0.0 (1.2)	0.04	−0.00 (1.2)
Missing *n* (%)	6 (0.4)	15 (0.7)		21 (0.6)
Systolic BP (mmHg) mean (SD)	138.5 (18.2)	133.6 (19.1)	< 0.001	135.5 (18.9)
Missing *n* (%)	0 (0)	1 (0.0)		1 (0.0)
Diastolic BP (mmHg) mean (SD)	84.9 (10.9)	82.48 (10.8)	< 0.001	83.4 (10.9)
Missing *n* (%)	0 (0)	1 (0.0)		1 (0.0)
Pulse pressure (mmHg) mean (SD)	53.7 (13.4)	51.1 (14.7)	< 0.001	52.1 (14.3)
Missing *n* (%)	0 (0)	1 (0.0)		1 (0.0)
Mean arterial pressure (mmHg) mean (SD)	102.76 (12.3)	99.51 (12.3)	< 0.001	100.8 (12.4)
Missing *n* (%)	0 (0)	1 (0.0)		1 (0.0)
Magnetic resonance scanner *n* (%)			0.47	
Skyra	634 (46.0)	1,003 (45.4)		1,637 (45.7)
Vida 1	675 (49.0)	1,126 (51.0)		1,801 (50.2)
Missing	69 (5.0)	78 (3.5)		147 (4.1)
Raw WMHV (mL) mean (SD)	4.3 (9.4)	3.1 (7.3)	< 0.001	3.6 (8.2)
Missing *n* (%)	115 (8.3)	154 (7.0)		269 (7.5)
Intracranial volume (mm^3^) mean (SD)	1,542,167.7 (116,266.4)	1,345,275.8 (98,176.7)	< 0.001	1,420,219.8 (142,315.2)
Missing *n* (%)	120 (8.7)	160 (7.2)		280 (7.8)
WMHV (Log/ICV adjusted) mean (SD)	0.8 (1.6)	0.9 (1.7)	0.38	0.8 (1.6)
Missing *n* (%)	156 (11.3)	227 (10.3)		383 (10.7)
Cardiovascular disease history *n* (%)	134 (9.7)	135 (6.1)	< 0.001	269 (7.5)
Clinical consensus cognitive status *n* (%)			< 0.001	
Cognitively normal	909 (66.0)	1,707 (77.3)		2,616 (73.0)
Mild cognitive impairment	353 (25.6)	379 (17.2)		732 (20.4)
Dementia	112 (8.1)	113 (5.1)		225 (6.3)
Undetermined	4 (0.3)	8 (0.4)		12 (0.3)

This table presents demographic, cardiometabolic, and white matter hyperintensities volume (log transformed and adjusted for intracranial volume, WMHV) characteristics of study participants, stratified by sex (Male versus Female). Continuous variables are displayed as mean (standard deviation), and categorical variables are shown as counts and percentages. Comparisons between males and females were conducted using *t*-tests for continuous variables and chi-square tests for categorical variables. *P*-values < 0.05 indicate significance.

Raw WMHV was greater in males than females (4.3 ± 9.4 mL vs. 3.1 ± 7.3 mL, *p* < 0.001). After ICV adjustment and logarithmic transformation, the mean WMHV did not differ significantly between sexes. Cognitive status also varied by sex (χ^2^, *p* < 0.001) with more females (77.3%) being cognitively normal; conversely, mild cognitive impairment (25.6% vs. 17.2%) and dementia (8.1% vs. 5.1%) were more frequent in males.

Both diabetes (β = 0.46, 95% CI 0.28–0.64; *p* < 0.001) and hypertension (β = 0.47, 0.30–0.64; *p* < 0.001) was associated with greater WMHV (Table 2). Poorer composite cardiometabolic risk scores were also associated with an increase in WMHV (β = 0.19, 0.12–0.26; *p* < 0.001). The significant interaction effect between sex and dyslipidemia and the main effect of obesity did not survive Bonferroni correction. Overall, no sex × risk-factor interactions reached the Bonferroni-adjusted significance level.

**TABLE 2:** Associations of cardiometabolic risk factors (Main effects and sex × risk-factor interactions) with ICV-adjusted white-matter-hyperintensity (WMH) volume.

Model	Term	β (95% CI)	*p*	*p* (Bonf.)
Diabetes	Main effect	**0.46 (0.28, 0.64)**	**< 0.001**	**< 0.001**
	Interaction	−0.17 (−0.39, 0.06)	0.16	1.00
Hypertension	Main effect	**0.47 (0.30, 0.64)**	**< 0.001**	**< 0.001**
	Interaction	−0.12 (−0.33, 0.10)	0.29	1.00
Dyslipidemia	Main effect	−0.09 (−0.26, 0.08)	0.30	1.00
	Interaction	**0.24 (0.02, 0.46)**	**0.03**	0.36
Obesity	Main effect	**0.17 (0.01, 0.33)**	**0.03**	0.40
	Interaction	−0.14 (−0.34, 0.06)	0.18	1.00
Tobacco	Main effect	**0.36 (0.11, 0.62)**	**0.005**	0.06
	Interaction	0.03 (−0.35, 0.42)	0.86	1.00
Risk score	Main effect	**0.19 (0.12, 0.26)**	**< 0.001**	**< 0.001**
	Interaction	−0.04 (−0.13, 0.04)	0.31	1.00

Linear models regressed natural-log–transformed WMH volume residuals (after adjustment for intracranial volume) on each risk factor, its interaction with sex, and the covariates age (years), education (years), and race/ethnicity (Non-Hispanic White, Non-Hispanic Black, and Hispanic). β values represent the change in log-WMH per unit (or presence vs. absence) of the risk factor; positive values indicate higher WMH burden. Confidence intervals (CI) and *p*-values are two-sided. A Bonferroni correction was applied across the 30 simultaneous tests in the table (α = 0.05/12 = 0.004); the resulting adjusted *p*-values (*p*-Bonf.) are shown in the last column. Effects meeting the nominal significance threshold (*p* < 0.05) are in bold; those that also surpass the Bonferroni-corrected threshold (*p*-Bonf. < 0.004) remain bold in the adjusted column.

Higher diastolic and MAP were robustly related to greater WMHV. Each 10 mmHg increase in DBP corresponded to an increase in WMHV (β = 0.18, 95% CI 0.11–0.26; *p* < 0.001), and each 10 mmHg increase in MAP was associated with greater WMHV (β = 0.14, 0.07–0.21; *p* < 0.001); both associations survived Bonferroni correction (Table 3). SBP showed only a nominal association with WMHV (β = 0.06, 0.02–0.11; *p* = 0.01), which did not remain significant after multiplicity adjustment. PP was not associated with WMHV (β = 0.01, −0.05 to 0.07; *p* = 0.82). No sex × BP interactions met the Bonferroni-adjusted threshold.

**TABLE 3:** Associations of blood-pressure metrics with ICV-adjusted white-matter hyperintensity (WMH) volume.

BP metric (per 10 mmHg) Model	Term	β (95% CI)	*p*	*p* (Bonf.)
Systolic BP	Main effect	**0.06 (0.02, 0.11)**	**0.01**	0.04
	Interaction	0.03 (−0.02, 0.09)	0.26	1.00
Diastolic BP	Main effect	**0.18 (0.11, 0.26)**	**< 0.001**	**< 0.001**
	Interaction	−0.08 (−0.17, 0.02)	0.11	0.85
Pulse pressure	Main effect	0.01 (−0.05, 0.07)	0.82	1.00
	Interaction	**0.11 (0.03, 0.18)**	**0.01**	0.03
Mean arterial pressure	Main effect	**0.14 (0.07, 0.21)**	**< 0.001**	**< 0.001**
	Interaction	−0.02 (−0.10, 0.07)	0.72	1.00

Linear models regressed natural-log–transformed WMH volume residuals (after adjustment for intracranial volume) on each blood-pressure (BP) metric, its interaction with sex, and the covariates age (years), education (years), and race/ethnicity (Non-Hispanic White, Non-Hispanic Black, and Hispanic). β coefficients express the change in log-WMH (adjusted for intracranial volume) per 10 mmHg increase in the BP metric; positive values indicate greater WMH burden. Confidence intervals (CI) and *p*-values are two-sided. A Bonferroni correction was applied to the eight *a priori* hypothesis tests (four BP main-effect terms and four sex-interaction terms): α = 0.05/8 = 0.006. The adjusted *p*-values (*p*-Bonf.) are shown in the last column. Results meeting the nominal threshold (*p* < 0.05) are in bold; those that also survive Bonferroni correction (*p*-Bonf. < 0.006) remain bold in the adjusted column.

After additional adjustment for diabetes, hypertension, dyslipidemia, obesity, and tobacco dependence (Table 4), DBP remained the sole BP parameter that was robustly related to WMHV (β = 0.14, 95% CI 0.07–0.22; *p* < 0.001).

**TABLE 4:** Associations of blood-pressure metrics with ICV-adjusted WMH volume after further adjustment for five CVD risk factors.

BP metric (per 10 mmHg) Model	Term	β (95% CI)	*p*	*p* (Bonf.)
Systolic BP	Main effect	0.03 (−0.02, 0.07)	0.22	1.00
	Interaction	0.03 (−0.03, 0.08)	0.30	1.00
Diastolic BP	Main effect	**0.14 (0.07, 0.22)**	**< 0.001**	**0.001**
	Interaction	−0.09 (−0.18, 0.01)	0.07	0.55
Pulse pressure	Main effect	−0.04 (−0.10, 0.02)	0.17	1.00
	Interaction	**0.11 (0.03, 0.18)**	**0.004**	0.03
Mean arterial pressure	Main effect	**0.10 (0.03, 0.17)**	**0.004**	0.04
	Interaction	−0.02 (−0.10, 0.06)	0.59	1.00

Linear models regressed natural-log–transformed WMH volume residuals (already adjusted for intracranial volume) on each blood-pressure (BP) metric, its interaction with sex, and the covariates age (years), education (years), race/ethnicity (non-Hispanic White, non-Hispanic Black, Hispanic), and the five cardiometabolic risk factors diabetes, hypertension, dyslipidemia, obesity, and tobacco use. β coefficients represent the change in log-WMH (adjusted for intracranial volume) for every 10-mmHg increase in the BP metric; positive values indicate greater WMH burden. Confidence intervals (CI) and *p*-values are two-sided. A Bonferroni correction was applied to the eight *a priori* hypothesis tests (four BP main-effect terms and four sex-interaction terms): α = 0.05/8 = 0.006. Adjusted *p*-values (*p*-Bonf.) are reported in the right-most column. Results meeting the nominal threshold (*p* < 0.05) are bold; those that also survive Bonferroni correction (*p*-Bonf. < 0.006) remain bold in the adjusted column.

## Discussion

The primary objective of this study was to identify whether common cardiometabolic conditions and blood-pressure traits explain white-matter hyperintensity burden differently in males versus females in a multi-ethnic cohort. In a large, racially ethnically diverse sample, we observed a clear pattern: diabetes, hypertension, an aggregate cardiometabolic risk score, and in particular, higher DBP were each linked to greater WMHV, whereas dyslipidemia, obesity, and tobacco dependence did not exhibit significant associations. Importantly, these associations did not differ by sex. Our findings suggest that the heaviest contributions to cerebral small-vessel injury come from shared metabolic and vascular pathways rather than sex-specific mechanisms in this specific population, reinforcing the need for aggressive management of cardiometabolic health to prevent WMHV exacerbation.

Mid-life hypertension is consistently linked to adverse neurodegenerative outcomes, whereas late-life hypertension demonstrates a less consistent relationship, sometimes even associating with protective outcomes (Glynn et al., 1999; Power et al., 2013). In our study, the absence of significant sex interactions for diabetes and hypertension might reflect the predominantly cognitively normal status of the cohort and the chronic effects of these conditions across a lifespan. Additionally, given the relatively older age of participants, vascular damage from diabetes and hypertension may have already reached a cumulative threshold, resulting in uniform effects between males and females. These findings of hypertension and diabetes confirm our results in an all-female subsample from HABS-HD (Hayes et al., 2025). Consistent with prior research, we observed a significant association between a composite cardiometabolic risk score and greater WMHV, though unlike some prior studies, this effect was not significantly stronger in females (Yau et al., 2024). The discrepancy in these findings are likely due to the Framingham cohort being predominantly NHW individuals whereas our cohort included both NHB and Hispanic, and we have previously shown that in a subcohort of females from HABS-HD, there are racial ethnic differences in risk factors and WMHV (Hayes et al., 2025).

Previous research indicated stronger associations of hypertension and elevated SBP with WMH progression in females, which persisted even after adjustment for menopausal status, hormone replacement therapy, and antihypertensive medications (Yau et al., 2024). We also found a significant association between SBP and WMHV; however, this association did not survive the Bonferroni correction. Yau et al. (2024) also found that higher DBP was associated with WMHV similar to our findings. This study is an extensive follow-up based on the future directions from Yau and colleagues in which they highlight the lack of racial ethnic diversity in their analytical sample which we hypothesize as the primary point for differences in findings.

A key strength of our investigation was the detailed assessment of individual blood pressure hemodynamics. Although both DBP and MAP were associated with increased WMHV in initial analyses, DBP emerged as the most robust predictor after comprehensive adjustment for cardiometabolic covariates, without significant sex interactions. These findings align with prior studies highlighting DBP as a critical blood pressure measure linked to WMHV, despite inconsistencies in the literature (Caunca et al., 2020; Gutierrez et al., 2015; Marcus et al., 2011; Yau et al., 2024). While studies such as the Strong Heart Study (Shibata et al., 2019) and the Cardiovascular Health Study (Longstreth et al., 1996) demonstrated strong SBP associations with WMHV or burden, other investigations (Yau et al., 2024) including ours, suggest that DBP may reflect particularly relevant aspects of cerebrovascular pathology. Our findings may be relatable to those uncovered in over 30,000 UK Biobank participants, where mid-life SBP and DBP were associated with greater WMHV, but, the relationship between DBP and WMHV was stronger in participants under age 50 (Wartolowska and Webb, 2021). Collectively, differences in study design, population characteristics (i.e., age and racial ethnic inclusion), and WMHV assessment methods likely contribute to differences in findings across studies.

PP, reflecting arterial stiffness and aging-related vascular impairment (Nation et al., 2012; Power et al., 2013), showed no significant association with WMHV in this study, contrary to prior evidence suggesting greater effects in postmenopausal women (Caughey et al., 2021). Conversely, MAP, a measure of overall vascular load (DeMers and Wachs, 2024) was robustly associated with WMHV but did not demonstrate sex differences, reinforcing the importance of comprehensive blood pressure control for cerebrovascular health in both sexes. However, these findings for MAP and WMHV were not independent of the cardiometabolic risk factors that we investigated.

These results underscore that blood pressure components differ in their associations with WMH burden, reflecting potentially distinct underlying vascular mechanisms. DBP appears particularly sensitive to chronic cerebrovascular damage, reinforcing the value of targeted interventions addressing this measurement specifically.

This study has several limitations warranting consideration. First, we lacked data on menopause, estrogen levels, and hormone therapy, factors influencing cerebrovascular outcomes in mid-life and older adult females. Future studies integrating detailed hormonal data in racially and ethnically diverse populations will help delineate these effects further (Yau et al., 2024). Second, the absence of visceral fat or waist-to-hip ratio measures limited the depth of obesity-related analyses. Third, racial ethnic specific stratification, which could elucidate intersectional effects of sex and racial ethnicity on WMHV, was beyond this study’s scope and warrants further investigation. Fourth, an imbalanced female-to-male ratio may influence the generalizability of sex-specific findings. Despite these limitations, our use of a PCA-derived cardiometabolic risk score offers advantages over traditional measures like the Framingham risk score, which can inflate cardiovascular risk estimates. Moreover, our detailed evaluation of multiple blood pressure components (SBP, DBP, PP, MAP) provides comprehensive insights into vascular contributions to WMHV and furthered their investigation by also controlling for cardiometabolic risk factors. Lastly, our analyses related on complete cases which assumes that data were missing completely at random, an assumption unlikely to hold in practice. While missingness was relatively low across analytic variables (< 10%), this approach may introduce bias if missingness was informative. Future studies incorporating multiple imputation could address this limitation.

Key strengths include our large, ethnically diverse cohort (> 3,000 participants) from the Southern US, which overcomes previous limitations highlighted in other studies. Additionally, our rigorous evaluation of cardiometabolic risks and blood pressure metrics advances our understanding of cerebrovascular pathology and offers robust evidence applicable to diverse populations.

In summary, our findings emphasize that cardiometabolic risk factors and specific blood pressure measures, particularly hypertension, diabetes, and DBP, significantly influences WMHV burden independent of sex in older adults from racially and ethnically diverse populations. While distinct blood pressure components contribute differently to cerebrovascular damage, sex-specific associations appear limited in this cohort. Future research should prioritize longitudinal assessments incorporating detailed menopausal status and hormonal data, stratify by racial ethnicity to investigate possible intersectional health disparities, and explore the role of specific obesity measures (e.g., visceral adiposity) in cerebrovascular health, especially among females. These directions will support tailored interventions addressing cardiometabolic risks and cerebrovascular disease prevention across diverse aging populations.

## Data Availability

Publicly available datasets were analyzed in this study. This data can be found here: https://apps.unthsc.edu/itr/.

## Footnotes

1
www.statisticalmodelling.de/lst.html

## The HABS-HD Study Team

HABS-HD MPIs: Sid E O’Bryant, Kristine Yaffe, Arthur Toga, Robert Rissman, and Leigh Johnson; and the HABS-HD Investigators: Meredith Braskie, Kevin King, James R Hall, Melissa Petersen, Raymond Palmer, Robert Barber, Yonggang Shi, Fan Zhang, Rajesh Nandy, Roderick McColl, David Mason, Bradley Christian, Nicole Phillips, Stephanie Large, Joe Lee, Badri Vardarajan, Monica Rivera Mindt, Amrita Cheema, Lisa Barnes, Mark Mapstone, Annie Cohen, Amy Kind, Ozioma Okonkwo, Raul Vintimilla, Zhengyang Zhou, Michael Donohue, Rema Raman, Matthew Borzage, Michelle Mielke, Beau Ances, Ganesh Babulal, Jorge Llibre-Guerra, Carl Hill and Rocky Vig.

## References

[B1] Appelman Y. van Rijn B. Ten Haaf M. Boersma E. Peters S. (2015). Sex differences in cardiovascular risk factors and disease prevention. *Atherosclerosis* 241 211–218. 10.1016/j.atherosclerosis.2015.01.027 25670232

[B2] Bonberg N. Wulms N. Dehghan-Nayyeri M. Berger K. Minnerup H. (2022). Sex-specific causes and consequences of white matter damage in a middle-aged cohort. *Front. Aging Neurosci.* 14:810296. 10.3389/fnagi.2022.810296 35645786 PMC9131069

[B3] Caughey M. Qiao Y. Meyer M. Palta P. Matsushita K. Tanaka H. (2021). Relationship between central artery stiffness, brain arterial dilation, and white matter hyperintensities in older adults: The ARIC study-brief report. *Arterioscler. Thromb. Vasc. Biol.* 41 2109–2116. 10.1161/ATVBAHA.120.315692 33882687 PMC8478115

[B4] Caunca M. Simonetto M. Cheung Y. Alperin N. Lee S. Elkind M. (2020). Diastolic blood pressure is associated with regional white matter lesion load: The northern manhattan study. *Stroke* 51 372–378. 10.1161/STROKEAHA.119.025139 31910743 PMC7219602

[B5] de Leeuw F. de Groot J. Oudkerk M. Witteman J. Hofman A. van Gijn J. (2002). Hypertension and cerebral white matter lesions in a prospective cohort study. *Brain* 125 765–772. 10.1093/brain/awf077 11912110

[B6] DeMers D. Wachs D. (2025). “Physiology, mean arterial pressure [Updated 2023 Apr 10],” in *StatPearls [Internet]*. Treasure Island, FL: StatPearls Publishing. Available online at: https://www.ncbi.nlm.nih.gov/books/NBK538226/30855814

[B7] Glynn R. Beckett L. Hebert L. Morris M. Scherr P. Evans D. (1999). Current and remote blood pressure and cognitive decline. *JAMA* 281 438–445. 10.1001/jama.281.5.438 9952204

[B8] Gutierrez J. Elkind M. Cheung K. Rundek T. Sacco R. Wright C. (2015). Pulsatile and steady components of blood pressure and subclinical cerebrovascular disease: The northern manhattan study. *J. Hypertens* 33 2115–2122. 10.1097/HJH.0000000000000686 26259124 PMC4871260

[B9] Hayes C. Odden M. Vintimilla R. Thorpe R. (2025). Racial ethnic variations in the cardiometabolic determinants and blood pressure of white matter hyperintensities among females-The HABS-HD Study. *Alzheimers Dement.* 21:e70327. 10.1002/alz.70327 40420353 PMC12106056

[B10] Hohman T. J. (2023). ADSP phenotype harmonization consortium. *Alzheimers Dement.* 19:e077713. 10.1002/alz.077713

[B11] Jeerakathil T. Wolf P. Beiser A. Massaro J. Seshadri S. D’Agostino R. (2004). *Stroke* risk profile predicts white matter hyperintensity volume: The Framingham Study. *Stroke* 35 1857–1861. 10.1161/01.STR.0000135226.53499.85 15218158

[B12] King K. Vintimilla R. Braskie M. Wei K. Hall J. Borzage M. (2022). Vascular risk profile and white matter hyperintensity volume among Mexican Americans and non-hispanic whites: The HABLE study. *Alzheimers Dement.* 14:e12263. 10.1002/dad2.12263 35229016 PMC8865739

[B13] Lee A. Sanchez D. Reyes-Dumeyer D. Brickman A. Lantigua R. Vardarajan B. (2023). Reliability and validity of self-reported vascular risk factors: Hypertension, diabetes, and heart disease, in a multi-ethnic community based study of aging and dementia. *J. Alzheimers Dis.* 95 275–285. 10.3233/JAD-230374 37483004 PMC10578288

[B14] Longstreth W. Manolio T. Arnold A. Burke G. Bryan N. Jungreis C. (1996). Clinical correlates of white matter findings on cranial magnetic resonance imaging of 3301 elderly people. The cardiovascular health study. *Stroke* 27 1274–1282. 10.1161/01.str.27.8.1274 8711786

[B15] Marcus J. Gardener H. Rundek T. Elkind M. Sacco R. Decarli C. (2011). Baseline and longitudinal increases in diastolic blood pressure are associated with greater white matter hyperintensity volume: The northern Manhattan study. *Stroke* 42 2639–2641. 10.1161/STROKEAHA.111.617571 21836088 PMC3189513

[B16] Morrison C. Dadar M. Manera A. Collins D. (2023). Racial differences in white matter hyperintensity burden in older adults. *Neurobiol. Aging* 122 112–119. 10.1016/j.neurobiolaging.2022.11.012 36543016

[B17] Nation D. Delano-Wood L. Bangen K. Wierenga C. Jak A. Hansen L. (2012). Antemortem pulse pressure elevation predicts cerebrovascular disease in autopsy-confirmed Alzheimer’s disease. *J. Alzheimers Dis.* 30 595–603. 10.3233/JAD-2012-111697 22451309 PMC3370943

[B18] O’Bryant S. Johnson L. Barber R. Braskie M. Christian B. Hall J. (2021). The health & aging brain among latino elders (HABLE) study methods and participant characteristics. Alzheimers Dement. *Diagn. Assess. Dis. Monit.* 13:e12202. 10.1002/dad2.12202 34189247 PMC8215806

[B19] O’Brien L. Ziegler D. Deutsch C. Frazier J. Herbert M. Locascio J. (2011). Statistical adjustments for brain size in volumetric neuroimaging studies: Some practical implications in methods. *Psychiatry Res.* 193 113–122. 10.1016/j.pscychresns.2011.01.007 21684724 PMC3510982

[B20] Patel V. Edison P. (2024). Cardiometabolic risk factors and neurodegeneration: A review of the mechanisms underlying diabetes, obesity and hypertension in Alzheimer’s disease. *J. Neurol. Neurosurg. Psychiatry* 95 581–589. 10.1136/jnnp-2023-332661 38290839 PMC11103343

[B21] Power M. Tchetgen E. Sparrow D. Schwartz J. Weisskopf M. (2013). Blood pressure and cognition: Factors that may account for their inconsistent association. *Epidemiology* 24 886–893. 10.1097/EDE.0b013e3182a7121c 24030502 PMC3818218

[B22] Prins N. Scheltens P. (2015). White matter hyperintensities, cognitive impairment and dementia: An update. *Nat. Rev. Neurol.* 11 157–165. 10.1038/nrneurol.2015.10 25686760

[B23] Samad Z. Wang T. Frazier C. Shah S. Dolor R. Newby L. (2008). Closing the gap: Treating hypertension in women. *Cardiol. Rev.* 16 305–313. 10.1097/CRD.0b013e31817f9350 18923234

[B24] Santos C. Snyder P. Wu W. Zhang M. Echeverria A. Alber J. (2017). Pathophysiologic relationship between Alzheimer’s disease, cerebrovascular disease, and cardiovascular risk: A review and synthesis. *Alzheimers Dement.* 7 69–87. 10.1016/j.dadm.2017.01.005 28275702 PMC5328683

[B25] Scharf E. Graff-Radford J. Przybelski S. Lesnick T. Mielke M. Knopman D. (2019). Cardiometabolic health and longitudinal progression of white matter hyperintensity: The mayo clinic study of aging. *Stroke* 50 3037–3044. 10.1161/STROKEAHA.119.025822 31510903 PMC6817406

[B26] Schmidt P. Gaser C. Arsic M. Buck D. Förschler A. Berthele A. (2012). An automated tool for detection of FLAIR-hyperintense white-matter lesions in multiple sclerosis. *Neuroimage* 59 3774–3783. 10.1016/j.neuroimage.2011.11.032 22119648

[B27] Shibata D. Suchy-Dicey A. Carty C. Madhyastha T. Ali T. Best L. (2019). Vascular risk factors and findings on brain MRI of Elderly adult American Indians: the strong heart study. *Neuroepidemiology* 52 173–180. 10.1159/000496343 30677776 PMC6986809

[B28] Vintimilla R. Hall J. Johnson L. Yaffe K. Toga A. O’Bryant S. (2021). Cardiovascular risk factors, white matter hyperintensities and executive function: Difference among Mexican Americans and non-hispanic whites. *Alzheimers Dement.* 17:e055620. 10.1002/dad2.12236 34692977 PMC8515357

[B29] Wartolowska K. Webb A. (2021). Midlife blood pressure is associated with the severity of white matter hyperintensities: Analysis of the UK Biobank cohort study. *Eur. Heart J.* 42 750–757. 10.1093/eurheartj/ehaa756 33238300 PMC7882359

[B30] Yau W. Scott M. Petrea R. Buckley R. Kojis D. Sperling R. (2024). Sex-specific vulnerabilities to subclinical vascular brain injury in early late-life: The framingham heart study. *Ann. Neurol.* 97 460–469. 10.1002/ana.27135 39540324 PMC12034097

