# Supplementary material for: Sex differences in the association of cardiometabolic risk scores and blood pressure measurements with white matter hyperintensities in diverse older adults—HABS-HD
Source: Front Aging Neurosci. 2025 Aug 4;17:1607646. doi: 10.3389/fnagi.2025.1607646 (PMC12358495; doi:10.3389/fnagi.2025.1607646)
Supplement: Supplementary file 1 [file Table_1.docx]

| **Supplemental Table 1: Variance Inflation Factors (VIFs) for Cardiometabolic, Behavioral, and Blood Pressure Models Evaluating ICV-Adjusted WMH** | | |
| --- | --- | --- |
| Model | Predictor | Adjusted VIF (GVIF^(1/(2*Df))) |
| Diabetes Model | Age | 1.06 |
|  | Education | 1.25 |
|  | Racial Ethnicity | 1.25 |
|  | Magnetic Resonance Imaging Scanner | 1.25 |
|  | Sex | 1.16 |
|  | Diabetes | 1.61 |
|  | Sex x Diabetes | 1.69 |
| Hypertension Model | Age | 1.08 |
|  | Education | 1.24 |
|  | Racial Ethnicity | 1.25 |
|  | Magnetic Resonance Imaging Scanner | 1.25 |
|  | Sex | 1.78 |
|  | Hypertension | 1.71 |
|  | Sex x Hypertension | 2.18 |
| Dyslipidemia Model | Age | 1.06 |
|  | Education | 1.24 |
|  | Racial Ethnicity | 1.24 |
|  | Magnetic Resonance Imaging Scanner | 1.25 |
|  | Sex | 1.81 |
|  | Dyslipidemia | 1.65 |
|  | Sex x Dyslipidemia | 2.21 |
| Obesity Model | Age | 1.07 |
|  | Education | 1.24 |
|  | Racial Ethnicity | 1.25 |
|  | Magnetic Resonance Imaging Scanner | 1.25 |
|  | Sex | 1.38 |
|  | Obesity | 1.65 |
|  | Sex x Obesity | 1.92 |
| Tobacco Model | Age | 1.06 |
|  | Education | 1.25 |
|  | Racial Ethnicity | 1.24 |
|  | Magnetic Resonance Imaging Scanner | 1.25 |
|  | Sex | 1.06 |
|  | Tobacco Dependence | 1.36 |
|  | Sex x Tobacco Dependence | 1.35 |
| Risk Score Model | Age | 1.06 |
|  | Education | 1.25 |
|  | Racial Ethnicity | 1.25 |
|  | Magnetic Resonance Imaging Scanner | 1.25 |
|  | Sex | 1.01 |
|  | Risk Score | 1.66 |
|  | Sex x RiskScore | 1.66 |
| Systolic Blood Pressure  (per 10mmHg) | Age | 1.07 |
|  | Education | 1.24 |
|  | Race | 1.25 |
|  | Magnetic Resonance Imaging Scanner | 1.25 |
|  | Sex | 7.62 |
|  | Systolic Blood Pressure | 1.72 |
|  | Sex x Systolic Blood Pressure | 7.55 |
| Diastolic Blood Pressure (per 10mmHg) | Age | 1.08 |
|  | Education | 1.24 |
|  | Race | 1.25 |
|  | Magnetic Resonance Imaging Scanner | 1.25 |
|  | Sex | 7.95 |
|  | Diastolic Blood Pressure | 1.66 |
|  | Sex x Diastolic Blood Pressure | 7.90 |
| Pulse Pressure (per 10mmHg) | Age | 1.12 |
|  | Education | 1.24 |
|  | Race | 1.25 |
|  | Magnetic Resonance Imaging Scanner | 1.25 |
|  | Sex | 4.01 |
|  | Pulse Pressure | 1.79 |
|  | Sex x Pulse Pressure | 4.13 |
| Mean Arterial Pressure (per 10mmHg) | Age | 1.06 |
|  | Education | 1.24 |
|  | Race | 1.25 |
|  | Magnetic Resonance Imaging Scanner | 1.25 |
|  | Sex | 8.53 |
|  | Mean Arterial Pressure | 1.67 |
|  | Sex x Mean Arterial Pressure | 8.44 |
| Systolic Blood Pressure  (per 10mmHg) Controlling for Risk Factors | Age | 1.10 |
|  | Education | 1.26 |
|  | Race | 1.27 |
|  | Magnetic Resonance Imaging Scanner | 1.25 |
|  | Sex | 7.63 |
|  | Diabetes | 1.06 |
|  | Hypertension | 1.19 |
|  | Dyslipidemia | 1.02 |
|  | Obesity | 1.05 |
|  | Tobacco Dependence | 1.03 |
|  | Systolic Blood Pressure | 1.80 |
|  | Sex x Systolic Blood Pressure | 7.56 |
| Diastolic Blood Pressure (per 10mmHg)  Controlling for Risk Factors | Age | 1.12 |
|  | Education | 1.26 |
|  | Race | 1.27 |
|  | Magnetic Resonance Imaging Scanner | 1.25 |
|  | Sex | 7.95 |
|  | Diabetes | 1.06 |
|  | Hypertension | 1.15 |
|  | Dyslipidemia | 1.02 |
|  | Obesity | 1.06 |
|  | Tobacco Dependence | 1.03 |
|  | Diastolic Blood Pressure | 1.72 |
|  | Sex x Diastolic Blood Pressure | 7.90 |
| Pulse Pressure (per 10mmHg)  Controlling for Risk Factors | Age | 1.14 |
|  | Education | 1.26 |
|  | Race | 1.27 |
|  | Magnetic Resonance Imaging Scanner | 1.25 |
|  | Sex | 4.02 |
|  | Diabetes | 1.06 |
|  | Hypertension | 1.13 |
|  | Dyslipidemia | 1.02 |
|  | Obesity | 1.05 |
|  | Tobacco Dependence | 1.03 |
|  | Pulse Pressure | 1.83 |
|  | Sex x Pulse Pressure | 4.14 |
| Mean Arterial Pressure (per 10mmHg)  Controlling for Risk Factors | Age | 1.10 |
|  | Education | 1.26 |
|  | Race | 1.27 |
|  | Magnetic Resonance Imaging Scanner | 1.25 |
|  | Sex | 8.54 |
|  | Diabetes | 1.06 |
|  | Hypertension | 1.19 |
|  | Dyslipidemia | 1.02 |
|  | Obesity | 1.05 |
|  | Tobacco Dependence | 1.03 |
|  | Mean Arterial Pressure | 1.75 |
|  | Sex x Mean Arterial Pressure | 8.45 |
| This table reports the adjusted variance inflation factors (GVIF^(1/(2*Df))) calculated for each predictor across all regression models used in the study. Models include cardiometabolic and behavioral risk factor models (Diabetes, Hypertension, Dyslipidemia, Obesity, Tobacco Dependence, and a composite Risk Score) as well as models for systolic blood pressure (SBP), diastolic blood pressure (DBP), pulse pressure (PP), and mean arterial pressure (MAP). Separate models examined blood pressure metrics alone and with adjustment for five cardiovascular risk factors. Interaction terms between sex and key exposures are reported where included. Adjusted VIF values >10 were prespecified as indicating problematic multicollinearity. All models demonstrated acceptable multicollinearity levels, with adjusted VIFs predominantly <2, and elevated VIFs for sex interaction terms reflecting structural correlation rather than problematic multicollinearity. | | |
