# Supplementary material for: Sex differences in the association of cardiometabolic risk scores and blood pressure measurements with white matter hyperintensities in diverse older adults—HABS-HD
Source: Front Aging Neurosci. 2025 Aug 4;17:1607646. doi: 10.3389/fnagi.2025.1607646 (PMC12358495; doi:10.3389/fnagi.2025.1607646)
Supplement: Supplementary file 2 [file Data_Sheet_1.zip › Supplemental_Figures-Legends.docx]

**Supplemental Figure 1. Impact of Outlier Removal on ICV-Adjusted WMH Regression Diagnostics**

**Legend.** Panel A displays the residuals versus fitted values from a linear model regressing natural‐log–transformed white matter hyperintensity volume (WMH<sub>log</sub>) on intracranial volume (ICV) in the full sample (n = 3,833). A small constant (1 × 10⁻⁶) was added to zero volumes before log‐transformation. Extreme residuals—defined as values lying more than 1.5 × IQR below the 25th or above the 75th percentile—were flagged as outliers (248 participants, 6% of the cohort). Panel B shows the same diagnostic plot after removing those outliers (remaining n = 3,585). In both panels, the red dashed line marks zero residual, and the blue dotted curve is a LOESS smoother.

**Supplemental Figure 2. Scree Plot of Principal Components for Harmonized Cardiometabolic Risk Scores**

**Legend.** The bar chart displays the percentage of total variance explained by the first five principal components derived from the 5 cardiometabolic risk factors (diabetes, hypertension, dyslipidemia, and tobacco dependence along with obesity defined by body mass index). Principal component 1 (PC1) accounts for 28.0% of the variance, PC2 for 20.3%, PC3 for 19.6%, PC4 for 16.6%, and PC5 for 15.4%. The steep drop in variance after PC1 indicates that a single latent factor captures the largest share of shared variance across the risk factors, with diminishing returns for subsequent components.

**Supplemental Figure 3. Residual‐vs‐Fitted Diagnostic Plots for WMH Regression Models**

**Legend.** Each panel displays the residuals versus fitted values for the multivariable linear regression models predicting natural‐log–transformed white matter hyperintensity volume (WMHV) residuals after adjustment for intracranial volume. **Risk‐Factor Models (Panels A–F):** Six separate models were fitted for each cardiometabolic risk factor—diabetes, hypertension, dyslipidemia, obesity, tobacco dependence—and for the composite cardiometabolic risk score derived by principal components analysis. In each model, WMHV was regressed on age (years), education (years), race/ethnicity (Non-Hispanic White, Non-Hispanic Black, Hispanic), neuroimaging scanner, the risk factor (or continuous risk score), sex, and their interaction (sex × risk factor).**Blood‐Pressure Models (Panels G–J):** Four models were fitted for systolic blood pressure (SBP), diastolic blood pressure (DBP), pulse pressure (PP), and mean arterial pressure (MAP), each scaled per 10 mmHg. A “base” model (covariates only) and an “extended” model (adjusting additionally for all five individual cardiometabolic risk factors) were both examined. In all panels, the dashed red line marks zero residual, and the dotted blue LOESS curve indicates any systematic deviation from zero. These plots confirm homoscedasticity and lack of strong nonlinear patterns, supporting the validity of the linear model assumptions.
