# Supplementary figures and images for: Sex differences in the association of cardiometabolic risk scores and blood pressure measurements with white matter hyperintensities in diverse older adults—HABS-HD
Source: Front Aging Neurosci. 2025 Aug 4;17:1607646. doi: 10.3389/fnagi.2025.1607646 (PMC12358495; doi:10.3389/fnagi.2025.1607646)

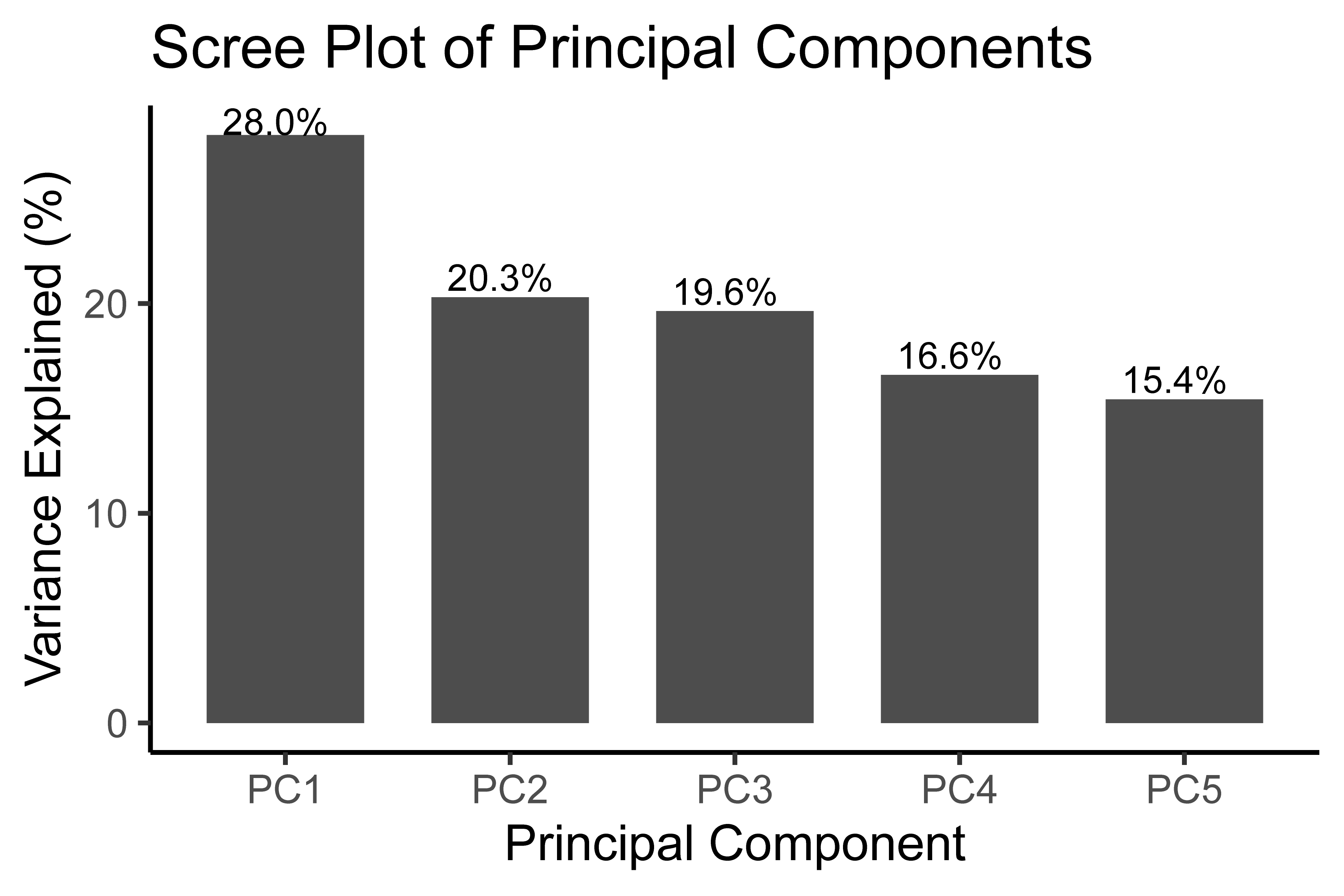

Supplement: Supplementary file 2 [file Data_Sheet_1.zip › Supp_Fig2.tif]

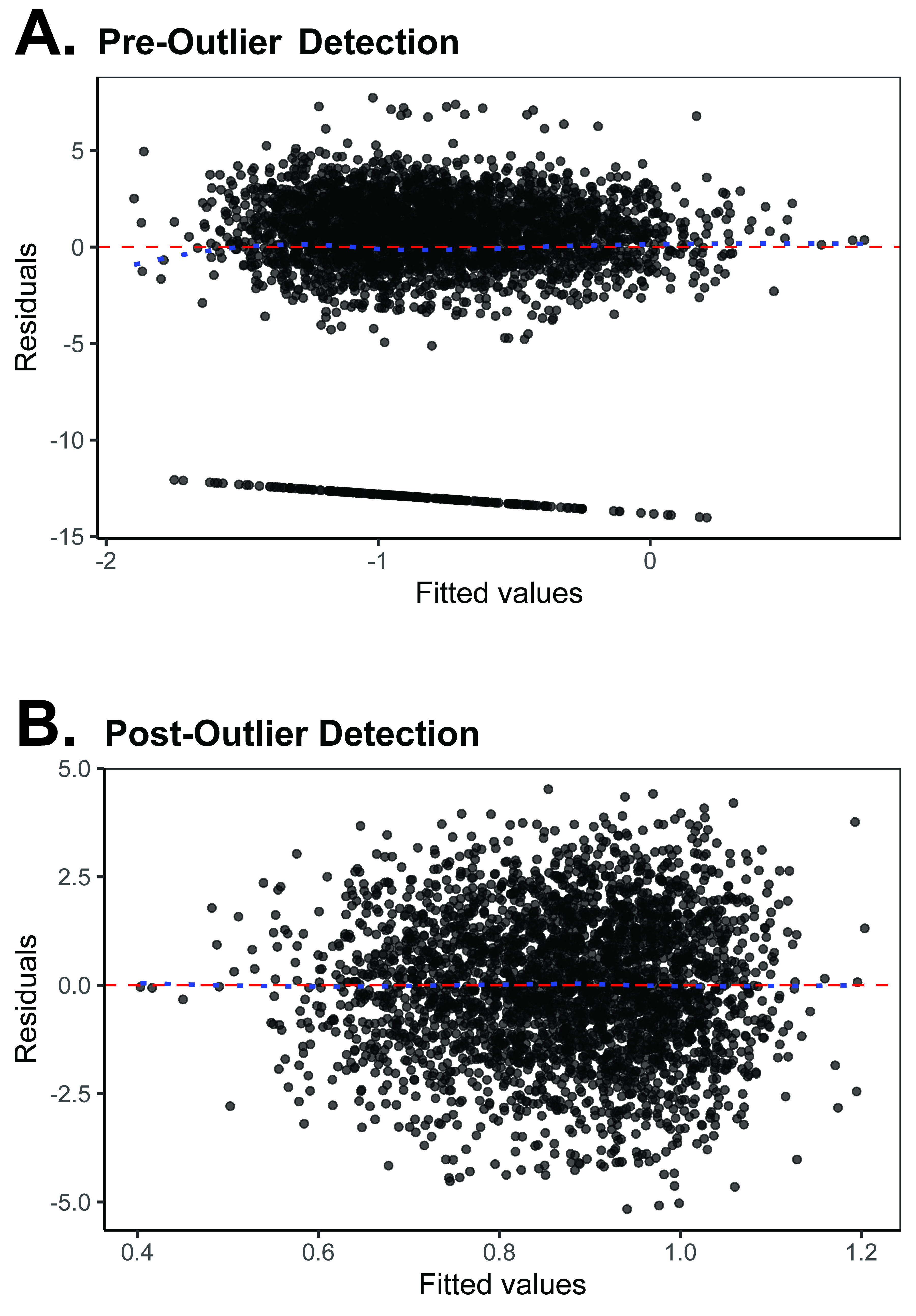

Supplement: Supplementary file 2 [file Data_Sheet_1.zip › Supp_Fig1.tif]
